# Temporal Fluctuation of Multidrug Resistant *Salmonella* Typhi Haplotypes in the Mekong River Delta Region of Vietnam

**DOI:** 10.1371/journal.pntd.0000929

**Published:** 2011-01-04

**Authors:** Kathryn E. Holt, Christiane Dolecek, Tran Thuy Chau, Pham Thanh Duy, Tran Thi Phi La, Nguyen Van Minh Hoang, Tran Vu Thieu Nga, James I. Campbell, Bui Huu Manh, Nguyen Van Vinh Chau, Tran Tinh Hien, Jeremy Farrar, Gordon Dougan, Stephen Baker

**Affiliations:** 1 The Wellcome Trust Sanger Institute, Wellcome Trust Genome Campus, Hinxton, Cambridge, United Kingdom; 2 Department of Microbiology and Immunology, The University of Melbourne, Melbourne, Australia; 3 The Hospital for Tropical Diseases, Wellcome Trust Major Overseas Programme, Oxford University Clinical Research Unit, Ho Chi Minh City, Vietnam; 4 Centre for Tropical Medicine, University of Oxford, Oxford, United Kingdom; 5 London School of Hygiene and Tropical Medicine, London, United Kingdom; 6 An Giang Provincial Hospital, My Binh, Long Xuyen, An Giang, Vietnam; 7 National Institute of Infectious and Tropical Diseases, Wellcome Trust Overseas Program, Oxford University Clinical Research Unit, Dong Da, Ha Noi, Vietnam; 8 The Hospital for Tropical Diseases, Ho Chi Minh City, Vietnam; The George Washington University Medical Center, United States of America

## Abstract

**Background:**

Typhoid fever remains a public health problem in Vietnam, with a significant burden in the Mekong River delta region. Typhoid fever is caused by the bacterial pathogen *Salmonella enterica* serovar Typhi (*S*. Typhi), which is frequently multidrug resistant with reduced susceptibility to fluoroquinolone-based drugs, the first choice for the treatment of typhoid fever. We used a GoldenGate (Illumina) assay to type 1,500 single nucleotide polymorphisms (SNPs) and analyse the genetic variation of *S*. Typhi isolated from 267 typhoid fever patients in the Mekong delta region participating in a randomized trial conducted between 2004 and 2005.

**Principal Findings:**

The population of *S*. Typhi circulating during the study was highly clonal, with 91% of isolates belonging to a single clonal complex of the *S*. Typhi H58 haplogroup. The patterns of disease were consistent with the presence of an endemic haplotype H58-C and a localised outbreak of *S*. Typhi haplotype H58-E2 in 2004. H58-E2-associated typhoid fever cases exhibited evidence of significant geo-spatial clustering along the Sông H 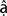
u branch of the Mekong River. Multidrug resistance was common in the established clone H58-C but not in the outbreak clone H58-E2, however all H58 *S*. Typhi were nalidixic acid resistant and carried a Ser83Phe amino acid substitution in the *gyrA* gene.

**Significance:**

The H58 haplogroup dominates *S*. Typhi populations in other endemic areas, but the population described here was more homogeneous than previously examined populations, and the dominant clonal complex (H58-C, -E1, -E2) observed in this study has not been detected outside Vietnam. IncHI1 plasmid-bearing *S*. Typhi H58-C was endemic during the study period whilst H58-E2, which rarely carried the plasmid, was only transient, suggesting a selective advantage for the plasmid. These data add insight into the outbreak dynamics and local molecular epidemiology of *S*. Typhi in southern Vietnam.

## Author Summary

Typhoid fever remains a serious public health issue in some parts of Vietnam, including the Mekong delta region. Typhoid is caused by the bacterium *Salmonella* Typhi, which is frequently multidrug resistant and shows reduced susceptibility to fluoroquinolone-based drugs. We assayed single nucleotide variation in the genomes of *S*. Typhi organisms isolated from 267 patients with typhoid fever in the Mekong delta between 2004 and 2005, and identified genetically distinct *S*. Typhi strains. We also detected the presence of genes or mutations that confer drug resistance in those strains. We found that the vast majority of typhoid cases were caused by one of two subgroups of H58 *S*. Typhi, referred to as H58-C and H58-E2. The H58-E2 group appeared to cause an outbreak in 2004, affecting patients living in a small zone near the Mekong River. The other group, H58-C, was present throughout the study period and affected patients living in a broader area of the Mekong River delta. Most of the H58-C strains were resistant to multiple drugs and carried a plasmid encoding multiple resistance genes. However very few H58-E2 strains were multidrug resistant, which may explain why the strain did not persist after the initial outbreak.

## Introduction

**Figure 1:**
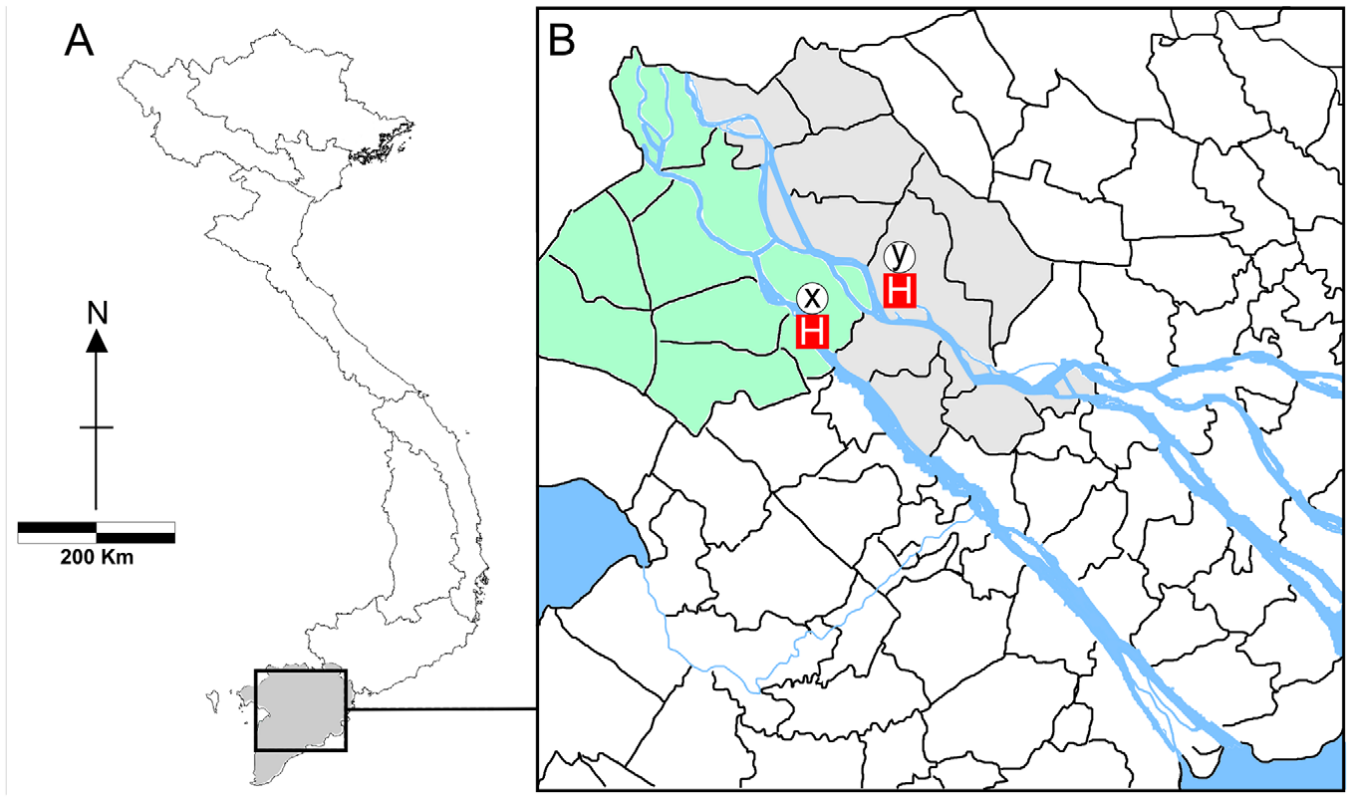
Location of hospitals in the Mekong river delta of Vietnam. (A) Map showing the 8 Vietnamese regions stretching from the Peoples Republic of China in the north to the Mekong river delta in the south. Highlighted in grey is Mekong river delta (Đ 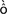
ng B 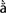
ng Sông C 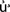
u Long) region, which is the southernmost of the eight regions and covers 40,000 km^2^. The dotted box corresponds to the area magnified in (B). (B) Map showing a ∼22,000 km^2^ of the Mekong river delta; highlighted are An Giang province (green) and Dong Thap province (grey). Also highlighted are the provincial hospitals of An Giang province (x) and Dong Thap (y). The direct distance between the two hospitals is 22.5 km.

The Mekong river delta is located in the south of Vietnam (Figure 1) in an area of 40,000 square kilometres (12% of Vietnam's land mass) and is home to over 20% of Vietnam's population. It is the area where the Mekong river divides into multiple channels and drains into the South China sea. The low-lying nature of the land and the seasonal fluctuation in water level make the region particularly vulnerable to flooding. The human-restricted disease typhoid fever is endemic to the Mekong delta region [1], [2], with a mean incidence of ∼80 cases per 100,000 people per year [1], [2], [3], [4]. *Salmonella* Typhi (*S*. Typhi), the bacterium causing typhoid fever, is transmitted human-to-human in areas with poor sanitation.

The first multidrug resistant (MDR; defined as resistance to chloramphenicol, ampicillin and co-trimoxazole) typhoid outbreak in Vietnam occurred in Kien Giang in the Mekong river delta in 1993 [5], and since then the fluoroquinolones have become the first choice for the treatment of typhoid fever. MDR *S*. Typhi is usually associated with self-transferrable IncHI1 plasmids carrying multiple resistance genes encoded within mobile genetic elements [6], [7], [8], [9], [10]. Between 1994 to 1998, over 80% of *S.* Typhi strains isolated in the Mekong delta region were reported to be MDR [11], and declined to approximately 50% between 2002 and 2004 [5], [11], [12]. This decline may have been catalysed by the change in treatment policy and the widespread use of fluoroquinolones (such as ciprofloxacin and ofloxacin), which are effective against MDR strains [13], [14].

While high-level resistance to fluoroquinolones remains uncommon in Vietnam and other endemic typhoid regions, there has been a sharp increase in the proportion of *S*. Typhi isolates that are resistant to nalidixic acid [11]. Nalidixic acid (Nal) is a quinolone antimicrobial (the precursor of fluoroquinolones) and the main mechanism for Nal resistance in *S.* Typhi is mutation of the DNA gyrase gene, *gyrA* [11], [15]. *S.* Typhi strains with Nal resistance-conferring mutations in the *gyrA* gene usually have elevated minimum inhibitory concentrations (MIC) to fluoroquinolone antibiotics such as ciprofloxacin (MIC ≥0.125 µg/ml) [16]. However, these organisms are not resistant according to CLSI guidelines, which are currently defined by MIC ≥4 µg/ml to ciprofloxacin [17]. Even though these strains are not classified as resistant, they are of clinical importance since typhoid patients infected with such strains respond less well to fluoroquinolone therapy [14], [15], [18], [19]. Such patients frequently have a protracted fever and an increased rate of relapse, compared to those infected with strains that do not have an elevated MIC to fluoroquinolones (MIC <0.125 µg/ml to ciprofloxacin and <0.25 µg/ml to ofloxacin) [15], [18], [19]. Resistance to Nal is therefore often used as a marker to predict how well a patient will respond to therapy with fluoroquinolones.

The incidence of typhoid fever has declined in Vietnam. Between 1991 and 2001 approximately 17,000 cases of typhoid fever (blood culture confirmed and syndromic cases) were reported annually through the Vietnamese national surveillance system [1], [2], while only 4,323 and 5,030 annual typhoid fever cases were reported in 2004 and 2005, respectively (Source: National Institute of Health and Epidemiology, Ministry of Health, Vietnam). However, 75% of these cases occurred in the Mekong delta [1], [2], likely associated with high population density and the propensity of the land to become saturated with floodwaters. In this region, the occurrence of *S*. Typhi isolates that are MDR and Nal resistant severely limits treatment options. More than 95% of *S.* Typhi isolated in the Mekong delta are now resistant to Nal, placing a considerable pressure on the effective use of fluoroquinolones [11], [12]. To compare alternative therapies for typhoid fever patients infected with strains that are MDR and Nal resistant, a randomized controlled trial comparing gatifloxacin (a newer 8-methoxy fluoroquinolone) and azithromycin (a macrolide) was conducted during 2004–2005 in the Mekong delta region [20]. Typhoid patients (adults and children) were recruited into the study at three hospitals in the south of Vietnam (details in Materials and Methods, locations are highlighted in Figure 1B). Here, we used a high-throughput single nucleotide polymorphism (SNP) typing assay to investigate the population structure of *S*. Typhi collected during the study [20], and to determine the genetic mechanisms of drug resistance in this *S*. Typhi population.

## Materials and Methods

### Ethics statement

The study was conducted according to the principles expressed in the Declaration of Helsinki and approved by the Institutional Review Board of the Hospital for Tropical Diseases and the Oxford Tropical Research Ethics Committee (OXTREC). All patients provided written informed consent for the collection of samples and subsequent analysis (written informed consent was provided by the parents or guardian of children under 18 years of age).

### Patient recruitment

*S*. Typhi isolates were collected during a multicenter clinical trial [20] conducted between January 2004 and December 2005 at (a) the Hospital for Tropical Diseases in Ho Chi Minh City (n = 10), (b) Dong Thap Provincial Hospital, Cao Lanh, Dong Thap province (n = 25) and (c) An Giang Provincial Hospital, Long Xuyen, An Giang province (n = 232). Locations of (b) and (c) are shown in Figure 1B. Adults and children over 6 months of age were eligible to be included in the study if they had clinically suspected or culture-confirmed uncomplicated typhoid fever and if fully informed written consent had been obtained. Patients were tested for typhoid carriage (via stool culture) during follow-up appointments at 1, 3 and 6 months after discharge from hospital. The 267 isolates presented in this study constitute nearly the full complement of 287 *S.* Typhi isolated from culture-confirmed typhoid patients enrolled in the trial; the recruitment flow for which is described in detail in [20].

### Antimicrobial susceptibility testing

Antimicrobial susceptibility testing was performed at the time of initial isolation by disc diffusion according to Clinical Laboratory Standards Institute (CLSI) guidelines [17]. Antimicrobial agents tested were: ampicillin, chloramphenicol, trimethoprim-sulfamethoxazole (co-trimoxazole), nalidixic acid, ofloxacin, ciprofloxacin and ceftriaxone (Oxoid, Basingstoke, UK). Minimum Inhibitory Concentrations (MICs) for amoxicillin, chloramphenicol, nalidixic acid, ofloxacin, ciprofloxacin, gatifloxacin, ceftriaxone and azithromycin were determined by E-test (AB Biodisk, Solna, Sweden). Multidrug resistance (MDR) of isolates was defined as resistance to chloramphenicol (MIC ≥32 µg/mL), ampicillin (MIC ≥32 µg/mL) and trimethoprim-sulfamethoxazole (MIC ≥8/152 µg/mL). Nalidixic acid resistance was defined by an MIC ≥32 µg/mL.

### Bacterial isolation and DNA preparation

After initial isolation, *S*. Typhi was stored at −70°C in a 20% glycerol solution until required for further analysis and DNA extraction. To revive frozen organisms, MacConkey and Xylose Lysine Decarboxylase (XLD) agar plates were inoculated from the glycerol solution and incubated at 37°C overnight. To ensure correct identification, colonies were checked using slide agglutination with serotype specific antisera (Vi, O9) and an irrelevant antisera as a negative control (O4) (Murex, Dartford, United Kingdom). Two mL of nutrient broth were inoculated with single *S*. Typhi colonies and incubated overnight. Overnight cultures were centrifuged and *S*. Typhi DNA was extracted using Wizard Genomic DNA Purification kit (Promega, USA) as recommended by the manufacturer's guidelines. DNA was stored at −20°C. DNA was quantified using the Quant-IT PicoGreen dsDNA Reagent and Kit (Invitrogen, UK). *S*. Typhi DNA concentrations were adjusted to 50 ng/mL and 250 ng of DNA were pipetted into 96-well plates. Each 96-well plate contained two isolates in duplicate and the sequenced *S*. Typhi isolate CT18 as control for assay reproducibility.

### Determination of chromosomal and plasmid haplotypes

The chromosomal haplotype of *S*. Typhi isolates was determined based on alleles present at 1,485 chromosomal SNP loci identified previously from genome-wide surveys [12], [21] and listed in [22], [23]. IncHI1 plasmid haplotypes were determined based on eight SNPs identified previously [22], [24] and resistance gene sequences were interrogated using additional oligonucleotide probes (listed in Table S1). All loci were interrogated using a GoldenGate custom oligonucleotide array according to the manufacturer's standard protocols (Illumina), as described previously [22], [23]. A maximum-likelihood phylogenetic tree based on chromosomal SNPs was constructed using the RAxML software [25].

### Statistical analysis

Clinical data were entered into an electronic database (Epi Info 2003, CDC, Atlanta, USA). For comparison of patient characteristics according to infecting *S*. Typhi haplotypes, Kruskal-Wallis tests were used for analysis of continuous variables (age, length of stay in hospital, fever clearance time) and logistic regression was used for categorical variables (presence of symptoms). Odds ratios were adjusted for duration of fever prior to admission and use of antibiotics prior to admission by including these variables in the logistic regression model. Where data was missing for a particular patient and variable, that patient was excluded from analysis of that variable (N≤35 patients). Two-tailed p-values are reported; statistical analysis was performed using the R package (http://www.r-project.org/).

### PCR amplification and sequencing of *gyrA* gene in *S*. Typhi

Oligonucleotide primers for the amplification of the quinolone resistance determining regions in the *S*. Typhi *gyrA* gene were as follows [11]: GYRA/P1 5′-TGTCCGAG ATGGCCTGAAGC-3′ and GYRA/P2 5′-TACCGTCATAAGTTATCCACG-3′. Predicted PCR amplicon size was 347 bp. PCR was performed under the following conditions; 30 cycles of: 92°C for 45 seconds, 45–62°C for 45 seconds and extension at 74°C for 1 minute, followed by a final extension step at 74°C for 2 minutes. PCR products were purified and directly sequenced using the CEQ DTCS - Quick Start Kit (Beckman Coulter, USA) and the CEQ 8000 capillary sequencer. The resulting DNA sequence was analyzed using CEQuence Investigator CEQ2000XL (Beckman Coulter, USA). All sequences were verified, aligned and manipulated using Bioedit software (http://www.mbio.ncsu.edu/BioEdit/bioedit.html) and compared to other *gyrA* sequences by BLASTn at NCBI.

### Spatial data collection and analysis

Patient addresses were recorded at the time of hospital admission. The latitude and longitude of the residences of typhoid fever patients (to the hamlet/village level) was assigned from the collected address data using 1/50,000 scale maps (Source: Cartographic Publishing House and VinaREN, Ministry of Natural Resources and Environment, Vietnam) and cross-checked using the websites http://www.basao.com.vn and http://ciren.vn. Location data was analysed using Quantum GIS version 1.4.0 (http://www.qgis.org/). Locations were colour-coded according to *S.* Typhi haplotype and clustering of specific haplotypes was calculated using the nearest-neighbour analysis function. Nearest-neighbour analysis examines the distances between each point and the closest point to it, and then compares these to expected values for a random sample of points from a CSR (complete spatial randomness) pattern. Significant clustering was inferred by Z-score value (standard normal variable) of less than 0; a positive score was interpreted as dispersion of locations.

## Results

### *S*. Typhi population structure

A recently developed typing system, based on the simultaneous interrogation of 1,485 *S*. Typhi chromosomal single nucleotide polymorphisms (SNPs) using a custom Illumina GoldenGate array [22], [23], was used to analyse each of the *S*. Typhi isolates. This approach facilitates the unequivocal assignment of isolates to haplotypes, allowing closely related strains to be distinguished phylogenetically based on single nucleotide changes. From 287 patients with culture confirmed typhoid fever recruited between January 2004 and December 2005 [20], 267 *S*. Typhi were available for SNP typing. These included 264 *S*. Typhi isolated from blood culture at admission [20] one relapse isolate and two faecal carriage isolates. A total of 24 *S*. Typhi (23 isolated from An Giang and one from Dong Thap, randomly distributed throughout the study period) were not available for SNP typing.

**Figure 2:**
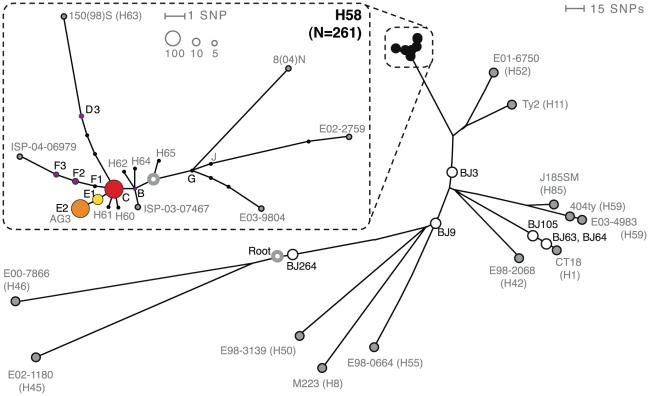
Phylogenetic distribution of *S.* Typhi isolates. Grey nodes represent control isolates (labelled by isolate code and haplotype group), unfilled grey circle indicates tree root, white nodes correspond to non-H58 *S*. Typhi isolated in this study (labelled with isolate code), black nodes show H58 isolates. Inset: zoom-in on the H58 haplogroup; grey nodes represent control isolates (labelled by isolate code or haplotype code), unfilled grey circle indicates tree root; coloured circles indicate nodes corresponding to H58 *S*. Typhi isolated in this study, node labels are as in the text, node colours are as in Figures 3–4, node sizes indicate the number of isolates on the scale as indicated by numbered circles.

A total of 261 *S*. Typhi isolates (98%) were of the common H58 haplogroup. The remaining isolates were of haplotypes H1 (isolates BJ105, BJ63, BJ64), H45 (isolate BJ264), H50 (isolate BJ9) and H52 (isolate BJ3; see Figure 2 and Table 1). The H58 *S*. Typhi isolates displayed variation at 10 SNP loci (detailed in [23]), which differentiated seven distinct sub-H58 haplotypes, shown in Figure 2. However, 242 (93%) of these isolates belonged to just three closely related H58 haplotypes, designated C, E1 and E2 in Figure 2 (numbers given in Table 1). The genome of *S*. Typhi strain AG3, isolated during the study (March 2004) from a typhoid fever patient living in An Giang province, was sequenced previously [21]. AG3 belongs to the H58-E2 haplotype, and the SNPs separating E2 from haplotypes E1 and C were originally identified by analysis of the AG3 genome. Therefore, the ability to differentiate within the cluster of 242 *S*. Typhi isolates was dependant on the inclusion of strain AG3 in the initial genome sequencing study used to identify SNP loci [21].

**Table 1:** Antimicrobial resistance pattern of *S*. Typhi haplotypes.

*S*. Typhi Haplotype	Total (% of all isolates)	Nal resistant (% of haplotype)	MDR (% of haplotype)	IncHI1 plasmid (% of haplotype)	MDR+Nal (% of haplotype)
**H58-C**	118* (44%)	117 (99%)	102 (86%)	92 (78%)	102 (86%)
**H58-E1**	15 (6%)	15 (100%)	15 (100%)	14 (93%)	15 (100%)
**H58-E2**	109* (41%)	109 (100%)	21 (19%)	17 (15%)	21 (19%)
**Other H58**	19 (7%)	16 (84%)	16 (84%)	16 (84%)	13 (68%)
**Non-H58**	6 (2%)	0 (0%)	0 (0%)	0 (0%)	0 (0%)
**Total**	267	257 (96%)	154 (58%)	139 (52%)	151 (56%)

All *S*. Typhi were isolated from blood culture, except two that were isolated from the stools of two chronic carriers.

*Includes one faecal carriage *S*. Typhi isolate. Nal  =  Nalidixic acid; MDR  =  multidrug resistant. Presence of IncHI1 plasmid was inferred from GoldenGate assay results (all isolates).

All but one *S*. Typhi isolated from the blood culture of patients admitted to An Giang Provincial hospital (231/232), as well as the two faecal *S*. Typhi strains isolated from chronic carriers in An Giang, belonged to the *S*. Typhi H58 haplogroup. The remaining *S*. Typhi isolate BJ264 (see Figure 2) was of the H45 haplotype and was isolated from a typhoid fever patient who was resident in neighbouring Can Tho province. One patient at An Giang Provincial hospital relapsed with symptoms of typhoid fever and had *S*. Typhi isolated from blood culture 11 days after the initial treatment (gatifloxacin) had been completed. The mother of the patient was found to be a chronic *S*. Typhi carrier. All three *S*. Typhi strains - the patient's admission and relapse blood culture isolates and the mother's faecal isolate - belonged to the *S*. Typhi H58-E2 subtype. The patient's isolates were both MDR and carried the IncHI1 ST6 plasmid (see below), whereas the mother's *S*. Typhi isolate was plasmid-free and susceptible to all first line antimicrobials at the time of isolation. All three isolates were Nal resistant but sensitive to gatifloxacin (MIC 0.19 mg/ml). Stool cultures were taken at 1 month (96% of patients), 3 months (93%) and 6 months (44% of follow-up. Chronic faecal carriage of *S*. Typhi was detected in only one trial patient. This was a MDR H58-C strain isolated from stool 6 months after treatment (with gatifloxacin), which was indistinguishable at all assayed loci from the patient's original blood culture isolate. Both isolates were Nal resistant but sensititve to gatifloxacin (MIC 0.19 mg/ml).

At Dong Thap Provincial Hospital, only 3 of the 25 *S*. Typhi isolates did not belong to the H58 haplogroup. Two H1 isolates (BJ63 and BJ64; Figure 2) were identical at all assayed loci and were isolated on consecutive days from two patients resident in Dong Thap. A third H1 strain (BJ105; Figure 2) differed from BJ63 and BJ64 at 16 chromosomal SNP loci and was isolated in Dong Thap 14 months after these isolates. Two siblings from Dong Thap province were admitted on consecutive days in 2004 and were both infected with MDR *S*. Typhi of the haplotype H58-C.

Of the ten *S*. Typhi strains isolated at the Hospital for Tropical Diseases in Ho Chi Minh City, eight were members of the H58 haplogroup, with patients resident in Ho Chi Minh City (n = 4), Long An (n = 1), Kien Giang (n = 2) and An Giang (n = 1) provinces, reflecting the larger catchment area of the hospital. The remaining two *S*. Typhi were of haplotypes H52 (BJ3) and H50 (BJ9) and were isolated from patients living in Binh Hoa province and Ho Chi Minh City, respectively.

There was no simple association between *S*. Typhi haplotype and patient age, length of stay in hospital, fever clearance time, vomiting, abdominal pain, hepatomegaly or relapse (Table 2). However, upon admission, patients infected with *S*. Typhi haplotype H58-E2 tended to report lower frequencies of diarrhoea and headache and higher frequencies of constipation compared to patients infected with other haplotypes, including H58-C (see Table 2).

**Table 2:** Selected characteristics of typhoid fever patients, based on baseline presentation history and outcomes.

Variable	*S.* Typhi H58-E2	Non-H58-E2 *S*. Typhi	*S*. Typhi H58-C	H58-E2 vs all other *S*. Typhi (95% CI)	p-value	Missing data
	n = 107	n = 157	n = 117			
**Age (yrs)**	11.9	12.2	12.7	Diff. −0.8 (−2.0,1.0)	0.83	0
**Time in hospital (days)**	13.9	13.7	13.8	Diff. 0.2 (−1.0,1.0)	0.74	0
**Fever clearance (hrs)**	116	115	121	Diff. 1 (−12,18)	0.70	2
**Constipation**	13.6%	5.8%	6.0%	OR 2.6 (1.1,6.1)	0.03	1
**(adjusted)**				OR 2.6 (1.1,6.0)	0.03	35
**Headache**	55.7%	70.1%	70.1%	OR 0.54 (0.32,0.90)	0.02	1
**(adjusted)**				OR 0.66 (0.40,1.09)	0.10	35
**Diarrhoea**	55.1%	72.6%	73.5%	OR 0.46 (0.28,0.78)	0.004	0
**(adjusted)**				OR 0.56 (0.34,0.93)	0.02	34
**Abdominal pain**	51.4%	56.1%	55.6%	OR 0.80	0.37	0
**(adjusted)**				OR 0.76 (0.47,1.24)	0.27	34
**Vomiting**	35.5%	35.5%	35.0%	OR 1.03	0.90	0
**(adjusted)**				OR 1.02 (0.61,1.71)	0.93	34
**Hepatomegaly**	58.5%	52.3%	51.3%	OR 1.35	0.23	2
**(adjusted)**				OR 1.30 (0.79,2.14)	0.30	35

Comparisons of selected characteristics among 264 typhoid fever patients (i.e. excluding carriage and relapse isolates). For continuous variables age, time in hospital and fever clearance, values shown are means and test statistic given is the difference in means (Diff.; mean value for H58-E2 *S*. Typhi – mean value for non-H58-E2 *S*. Typhi). Other variables indicate frequency of symptoms self-reported at time of admission and of clinician-diagnosed hepatomegaly; test statistic is odds ratio (OR) for H58-E2 *S*. Typhi vs non-H58-E2 *S*. Typhi; both crude OR and adjusted OR are reported (adjusted for duration of fever prior to admission and use of antibiotics prior to admission, using logistic regression). All comparisons shown are for patients infected with H58-E2 *S*. Typhi vs those infected with other *S*. Typhi haplotypes (including H58-C and others); 95% confidence intervals (CI) are given in brackets.

### Plasmids and antimicrobial resistance

The GoldenGate assay incorporated probes targeting IncHI1 plasmid sequences, allowing for detection of the presence of IncHI1 plasmid within the genomic DNA extracted from each *S*. Typhi isolate. The assay indicated that a total of 139 *S*. Typhi isolates harboured an IncHI1 plasmid. All plasmids were of the IncHI1 ST6 sequence type [24] and all plasmid-bearing isolates belonged to the *S*. Typhi H58 haplogroup (see Table 1). The MDR IncHI1 plasmid was more common among H58-C isolates than H58-E2 isolates (86% vs 19%, see Table 1). Of the 139 *S*. Typhi isolates giving positive signals for IncHI1 SNP loci, 137 (99%) were classified as MDR by antimicrobial susceptibility testing conducted at the time of isolation. One other IncHI1-positive isolate tested positive by GoldenGate assay for the genes *sul1*, *sul2*, *dfrA7*, *tetACDR*, *strAB*, *bla* and *cat* (resistance genes; functions outlined in Table S1) like the MDR isolates, yet had low MICs for chloramphenicol, ampicillin and trimethoprim-sulfamethoxazole. An additional *S*. Typhi isolate, BJ5, was resistant to ampicillin and trimethoprim-sulfamethoxazole but sensitive to chloramphenicol. This was consistent with GoldenGate assay results, which gave positive signals for the *repC* replication initiation gene of IncHI1, resistance genes *strAB*, *bla*, *sul1*, *sul2*, *dfrA7*, but no signal for sequences from the *cat* gene encoding chloramphenicol resistance. A further 17 *S*. Typhi isolates were recorded as MDR according to their antimicrobial susceptibility pattern at the time of isolation, but did not test positive for IncHI1 plasmid loci. This likely reflects loss of the IncHI1 plasmid in culture or storage between the time of isolation and DNA extraction. The MDR status of the infecting *S*. Typhi isolate was not associated with fever clearance time (p = 0.3, two-sided T-test) or treatment failure (p = 0.18, Chi^2^ test).

A total of 257 *S*. Typhi isolates were resistant to nalidixic acid (Nal). All of these isolates belonged to the H58 haplogroup (Table 1) and all were susceptible to gatifloxacin, ciprofloxacin and ofloxacin according to current CLSI guidelines [17]. *S*. Typhi haplotypes H58-C, H58-E1 and H58-E2 were uniformly resistant to Nal, with the exception of a single H58-C isolate which had an intermediate MIC of 28 µg/mL (resistance defined as MIC ≥32 µg/mL). The sequenced H58-E2 isolate AG3 harbours a mutation changing serine (TCC) to phenylalanine (TTC) at codon 83 in the *gyrA* gene (GyrA-Ser83Phe) [21], which is known to confer resistance to Nal [26]. In the present study we sequenced the *gyrA* gene in 223 of the Nal resistant isolates (87%) and found the same GyrA-Ser83Phe amino acid substitution in all isolates tested.

### Spatial and temporal distribution

**Figure 3:**
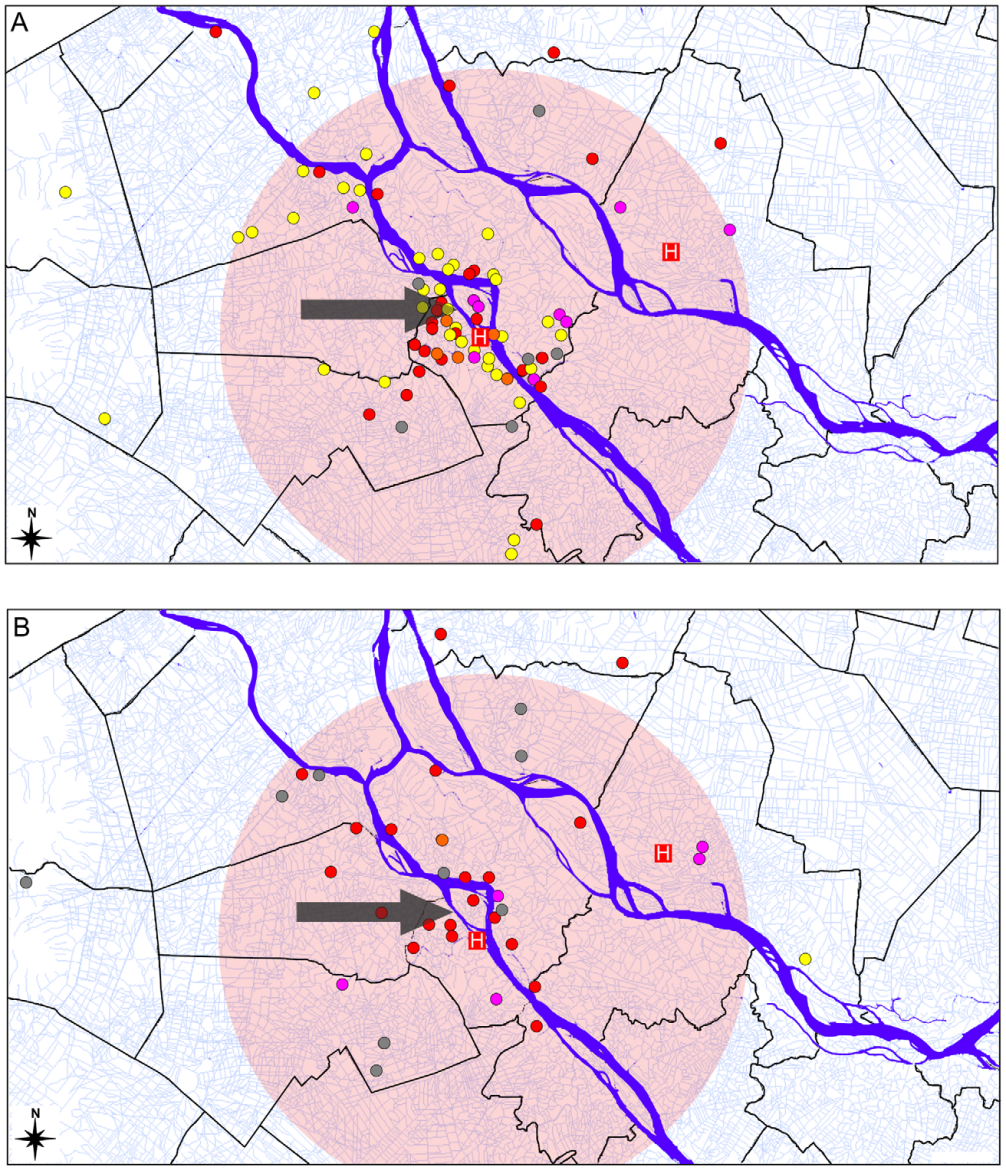
Spatial distribution of *S.* Typhi isolates by haplotype and year. The spatial distribution of *S*. Typhi haplotypes surrounding An Giang provincial hospital in (A) 2004 and (B) 2005. Each point corresponds to the residential location of a typhoid fever patient; colour indicates the haplotype of the *S*. Typhi isolate (with or without plasmid): dark orange  =  H58-E2 with MDR plasmid, light orange  =  H58-E2 without MDR plasmid, dark red  =  H58-C with MDR plasmid, pink, H58-C with MDR plasmid, grey  =  other *S.* Typhi haplotypes. Locations of the hospitals are indicated by a white cross on a red background; pink circle indicates a radius of 15 km from An Giang Provincial Hospital; arrow indicates the Sông H 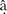
u branch of the Mekong river.

Figure 3 shows the spatial distribution of the residences of 160 typhoid patients (this information was not available for the remaining patients). Of the patients admitted at An Giang Provincial Hospital and Dong Thap Provincial Hospital, sufficient address detail to allow for assignment of latitude and longitude was provided in 61% and 73% of cases, respectively. This represents 50% and 20% of all blood culture confirmed typhoid fever patients at An Giang Provincial Hospital and Dong Thap Provincial Hospital, respectively, during 2004–2005. In An Giang, patients' homes clustered around the An Giang Provincial Hospital, but also around the Sông H 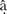
u branch of the Mekong river (see Figure 3). Most *S*. Typhi isolated from patients living near this point in An Giang province were of the H58-E2 haplotype (orange in Figure 3), and this group demonstrated significant clustering using nearest-neighbour analysis (n = 57, Z-score  = −14.145). In contrast, *S*. Typhi of the H58-C haplotype were isolated relatively frequently in neighbouring provinces and had a more sporadic clustering pattern (red in Figure 3). While isolates from An Giang Provincial Hospital are overrepresented in this spatial analysis, the apparent increase in typhoid density in An Giang is consistent with total Typhi isolation rates at the two hospitals during the study period (284 at An Giang Provincial Hospital and 90 at Dong Thap Provincial Hospital).

**Figure 4:**
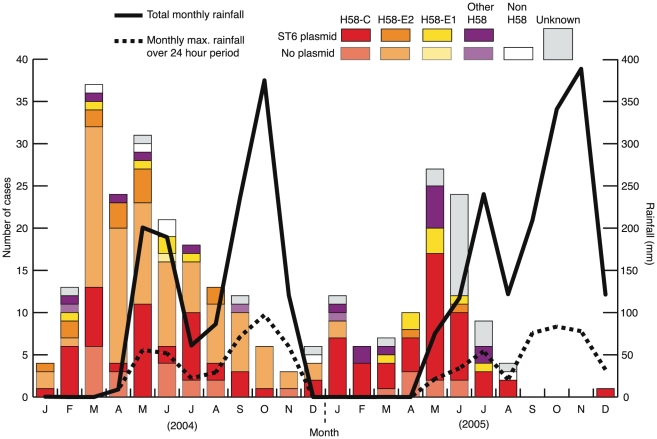
Monthly incidence of typhoid cases by haplotype. Bar heights indicate the total number of *S*. Typhi isolated each month during the study, according to the scale given on the left-hand y-axis; colours indicate the combination of *S.* Typhi haplotype and presence of IncHI1 ST6 plasmid as given in the legend. Solid black line  =  total rainfall each month recorded in An Giang, dashed line  =  maximum rainfall occurring in a 24 h period during each month in An Giang; rainfall scale is shown on the right-hand y-axis.

The temporal distribution of *S*. Typhi haplotypes over 2004 and 2005 is shown in Figure 4. Typhoid fever cases peaked just prior to the onset of the wet season in each year, as has been observed previously in this region [1], [3] (see monthly rainfall, solid line in Figure 4). In 2004, H58-E2 and H58-C were both prevalent (62 C, red in Figure 4; 103 E2, orange in Figure 4), whereas few isolates of H58-E2 Typhi were observed during 2005 (55 C, 4 E2; see Figure 4). The decline of H58-E2 may be associated with selection for the IncHI1 MDR plasmid, which was much more common in H58-C (Table 1). As Figure 4 highlights, the majority of isolates collected during the second season were MDR and carried the IncHI1 plasmid ST6.

## Discussion

Our data show the vast majority of *S*. Typhi isolates (n = 261, 98%) isolated from the Mekong delta during the two-year study period belonged to the H58 haplogroup. Furthermore, 91% of isolates (n = 242) belonged to a single clonal complex of *S*. Typhi H58 (nodes C, E1, E2 shown in Figure 2), demonstrating remarkable homogeneity in the *S.* Typhi population in this location during the study period. The observed level of clonal dominance is greater than that observed in previous haplotyping studies of local *S*. Typhi populations. Among 54 *S.* Typhi isolates from Jakarta, Indonesia between 1975 and 2005, a total of nine haplotypes were detected, with the dominant H59 haplotype accounting for 53% of isolates; the next most frequent haplotype was genetically distant from H59 and comprised 24% of isolates [27]. In Kathmandu, Nepal, a collection of *S*. Typhi isolated from children hospitalised with typhoid fever in 2005–2006 was dominated by the H58-G haplotype (66%) but the distant H42 haplotype was also present at high frequency (19%) [23]. Among *S*. Typhi isolated between 2001 and 2008 in Nairobi, Kenya, 87% were H58, although two distinct subtypes (nodes B and J, see Figure 2) were co-circulating at equally high frequencies (>40% each) between 2004 and 2008 [22]. *S*. Typhi H58 nodes B and J (co-circulating in Nairobi) represent distinct lineages, each acquiring unique SNPs since the last common ancestor of H58 (Figure 2). However H58 nodes C, E1 and E2, which account for 91% of isolates in this study in Vietnam, are closely related and formed a tight clonal complex differentiated by just two SNPs (Figure 2). Thus the overall level of clonality of the *S.* Typhi population analysed in this study was unexpectedly high. The clonal complex comprising H58-C, -E1 and -E2 was not detected in study populations in Nepal and Kenya where the same SNP typing method was used [22], [23], suggesting it may have arisen locally in Vietnam.

Despite the genetic homogeneity we observed, the availability of whole-genome sequence data for *S.* Typhi H58-E2 isolate AG3 [21], collected during the study, allowed us to differentiate closely related organisms within the H58 group. Just two SNPs identified in strain AG3 subdivided the homogeneous group into three nodes C, E1 and E2 (Figure 2), of which two (C and E2) were dominant (>40% each). Isolates belonging to the H58-C node were present at a constant rate during the two years of the study (62 isolates in 2004 and 55 in 2005). However, isolates belonging to the H58-E2 node were common during 2004 (103 isolates), yet were virtually undetected in 2005 (3 isolates). This change in both haplotype distribution and total number of typhoid cases from 2004 to 2005 is striking, and suggests an outbreak caused by *S*. Typhi H58-E2 during 2004. We additionally found that H58-C strains had a much stronger association with the ST6 IncHI1 MDR plasmid than H58-E2 (Table 1). We speculate that the persistence of H58-C strains and the corresponding disappearance of H58-E2 may be associated with a competitive phenotypic advantage conferred by the IncHI1 MDR plasmid. However, it is important to remember that node C is a precursor of node E2 and we can only differentiate E2 from C because we had whole genome sequence data for an H58-E2 strain from which to identify SNPs [21]. Thus the population of *S*. Typhi isolates assigned to node C by our SNP typing assay may be more diverse than that assigned to H58-E2. It is also important to note that since our data covers just two years, it is possible that any competitive advantage of H58-C strains may be short-lived and there is no evidence for long-term replacement of H58-E2.

We identified two cases of chronic faecal carriage of *S*. Typhi during the course of the study, one in a patient's relative and one in a patient after 6 months of follow-up. This underlines the importance of screening procedures to identify carriers and effective treatment to eliminate carriage and reduce transmission. The faecal *S*. Typhi isolates were of the dominant H58-E2 and H58-C haplotypes, respectively. In a previous case-control study performed in the Mekong delta, close contact with a patient with typhoid fever was significantly associated with developing the disease compared to hospital controls (adjusted odds ratio (OR)  = 5.2, 95% confidence interval (95% CI) [1.7, 15.9]) or community controls (adjusted OR  = 11.9, 95% CI [2.3, 60.7]) [28].

We were able to collect residential location data from 160 typhoid patients (61%). While typhoid patients reporting to An Giang Provincial Hospital are overrepresented in this data set (50% of all confirmed cases vs. 20% of all confirmed cases at Dong Thap Hospital), the apparent clustering in An Giang (Figure 3) is consistent with the overall isolation rates at the two hospitals, which during the study period was more than three times higher at An Giang Provincial Hospital than Dong Thap Hospital. The data set provides roughly equal representation of patients infected with *S*. Typhi H58-C (65%) and H58-E2 (62%), thus any differences in spatial distribution between patients presenting at the different hospitals should not affect the differences between spatial distribution of these haplotypes. Spatial clustering of *S*. Typhi H58-E2 was evident particularly around the Sông H 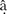
u branch of the Mekong river, while other *S*. Typhi haplotypes were more broadly distributed (Figure 4). The spatial clustering of H58-E2 *S.* Typhi further supports a localised outbreak in 2004 caused by these isolates. In contrast, the broader spatial and temporal distribution of *S*. Typhi H58-C during the study suggests it may be well established in the community and can persist over longer distances and time periods.

We also observed that some symptoms reported by patients infected with H58-E2 *S*. Typhi differed from those infected with other *S*. Typhi haplotypes (Table 2). After adjusting for antibiotic use and duration of fever prior to admission, patients infected with H58-E2 *S*. Typhi were more likely to report diarrhoea and headache compared with other *S*. Typhi haplotypes (OR  = 0.56, 95% CI [0.34, 0.93] and OR  = 0.66, 95% CI [0.40, 1.09], respectively), but were more commonly associated with constipation (OR  = 2.6, 95% CI [1.1, 6.0]). This suggests there may be some phenotypic differences between H58-E2 and other *S*. Typhi with respect to disease, however these were post-hoc analyses and no adjustments were made for multiple comparisons, hence these associations should be interpreted with caution. However if confirmed in subsequent prospective studies, it would be of interest to know whether these phenotypic characteristic were associated with specific mutations in H58-E2 *S*. Typhi. The two SNPs differentiating the E2 node from E1 and C are both synonymous mutations (C->T in *melA* (nt 315); G->A in *rbsA* (nt 576)) and our earlier analysis of the AG3 sequence data detected no phage insertions and no large deletions that were not also detected in other sequenced H58 isolates [21]. However, we were unable to verify if other single-base insertions or deletions were present, which may result in gene inactivation with corresponding phenotypic effects.

Patterns of antimicrobial resistance of *S.* Typhi tend to vary markedly between different typhoid-endemic regions. In this present work, as in the recent study of Kenyan isolates [22], there were high rates of MDR associated with IncHI1 ST6 plasmids among strains of the *S*. Typhi H58 haplogroup. This suggests that the presence of the plasmid may contribute to the success of the dominant *S*. Typhi haplotypes, and the results of our study corroborate this hypothesis. The *S*. Typhi H58-E2 subtype (which was generally not associated with a plasmid) was only transient, while the H58-C subtype (which was more commonly associated with the IncHI1 MDR plasmid) was present in 2004 and 2005 in southern Vietnam. In Kenya, almost all isolates of the dominant H58 haplotypes carried the MDR plasmid, while the plasmid-free H58-G subtype was only detected twice [22]. All the H58 isolates analysed in the present study were resistant to Nal, conferred by an identical mutation in *gyrA*. This is consistent with previous studies reporting strong associations between the Nal resistance phenotype and the H58 haplogroup of *S.* Typhi [12], [22], [23]. The presence of the same mutation conferring Nal resistance in all isolates of H58-C, -E1 and -E2 suggests this mutation may have arisen in the common ancestor of this clonal complex, perhaps *in situ* in the Mekong delta region, and its continued presence is likely maintained by selective pressure exerted by the use of fluoroquinolones.

### Conclusions

During 2004–2005, typhoid in the Mekong river delta region of Vietnam was almost exclusively caused by a single Nal-resistant clonal complex of *S*. Typhi. This reflects a higher level of clonality than observed in other localised *S*. Typhi populations studied to date, which may be indicative of higher transmission rates in this location. The high level of Nal resistance and multidrug resistance, frequently in the same strains, is concerning and continues to pose problems for the successful treatment of typhoid fever.

## Supporting Information

Checklist S1 Strobe Checklist
(0.09 MB DOC)

Table S1 IncHI1 plasmid and resistance-associated gene targets.
(0.03 MB XLS)

Translation S1 Translation of the abstract into Vietnamese by Nga Tran Vu Thieu.
(0.09 MB RTF)
