# Supplementary material for: Temporal Fluctuation of Multidrug Resistant Salmonella Typhi Haplotypes in the Mekong River Delta Region of Vietnam
Source: PLoS Negl Trop Dis. 2011 Jan 4;5(1):e929. doi: 10.1371/journal.pntd.0000929 (PMC3014949; doi:10.1371/journal.pntd.0000929)
Supplement: Checklist S1 — Strobe Checklist (0.09 MB DOC) [file pntd.0000929.s001.doc]

STROBE Statement—checklist of items that should be included in reports of observational studies

|  | Item No | Recommendation |
| --- | --- | --- |
| **Title and abstract** | 1 | (*a*) Indicate the study’s design with a commonly used term in the title or the abstract  Abstract, p2 |
|  |  | (*b*) Provide in the abstract an informative and balanced summary of what was done and what was found Abstract, p2-3 |
| Introduction | | |
| Background/rationale | 2 | Explain the scientific background and rationale for the investigation being reported  Intro, p1-7 |
| Objectives | 3 | State specific objectives, including any prespecified hypotheses  Intro, p7 |
| Methods | | |
| Study design | 4 | Present key elements of study design early in the paper  Methods, p8 |
| Setting | 5 | Describe the setting, locations, and relevant dates, including periods of recruitment, exposure, follow-up, and data collection  Methods, p8-11; Results, p12 |
| Participants | 6 | (*a*) *Cohort study*—Give the eligibility criteria, and the sources and methods of selection of participants. Describe methods of follow-up  Methods, p8  *Case-control study*—Give the eligibility criteria, and the sources and methods of case ascertainment and control selection. Give the rationale for the choice of cases and controls  *Cross-sectional study*—Give the eligibility criteria, and the sources and methods of selection of participants |
|  |  | (*b*) *Cohort study*—For matched studies, give matching criteria and number of exposed and unexposed  N/A  *Case-control study*—For matched studies, give matching criteria and the number of controls per case |
| Variables | 7 | Clearly define all outcomes, exposures, predictors, potential confounders, and effect modifiers. Give diagnostic criteria, if applicable  Typhoid fever case definition – positive culture of S. Typhi; Methods p8  Clinical symptoms at admission are self-reported only; Results p14  MICs for antimicrobial resistance; Methods p9  Multidrug resistance (MDR) definition; Methods p9 |
| Data sources/ measurement | 8* | For each variable of interest, give sources of data and details of methods of assessment (measurement). Describe comparability of assessment methods if there is more than one group  Haplotype determination is described in detail in Methods p10; target loci are given in cited papers and supplementary table 1 |
| Bias | 9 | Describe any efforts to address potential sources of bias |
| Study size | 10 | Explain how the study size was arrived at  The isolates analysed in this manuscript were taken from a previous randomized control trial, the study design for the trial is described in ref 20 as cited throughout the text. All available isolates were included, as stated in Methods p8. |
| Quantitative variables | 11 | Explain how quantitative variables were handled in the analyses. If applicable, describe which groupings were chosen and why  N/A, quantitative variables not grouped. |
| Statistical methods | 12 | (*a*) Describe all statistical methods, including those used to control for confounding  Methods p10-11 |
|  |  | (*b*) Describe any methods used to examine subgroups and interactions  Methods p10 |
|  |  | (*c*) Explain how missing data were addressed  Methods p10-11 |
|  |  | (*d*) *Cohort study*—If applicable, explain how loss to follow-up was addressed  *Case-control study*—If applicable, explain how matching of cases and controls was addressed  *Cross-sectional study*—If applicable, describe analytical methods taking account of sampling strategy  Methods p10-11 |
|  |  | (*e*) Describe any sensitivity analyses  N/A |

Continued on next page

| Results | | |
| --- | --- | --- |
| Participants | 13* | (a) Report numbers of individuals at each stage of study—eg numbers potentially eligible, examined for eligibility, confirmed eligible, included in the study, completing follow-up, and analysed  Described in detail in ref 20 |
|  |  | (b) Give reasons for non-participation at each stage  Described in detail in ref 20 |
|  |  | (c) Consider use of a flow diagram  Described in detail in ref 20 |
| Descriptive data | 14* | (a) Give characteristics of study participants (eg demographic, clinical, social) and information on exposures and potential confounders  Described in detail in ref 20  Age and relevant clinical symptoms are given in Table 2 |
|  |  | (b) Indicate number of participants with missing data for each variable of interest  Table 2 |
|  |  | (c) *Cohort study*—Summarise follow-up time (eg, average and total amount)  Results, p13 |
| Outcome data | 15* | *Cohort study*—Report numbers of outcome events or summary measures over time  Results, p17; Figure 4 |
|  |  | *Case-control study—*Report numbers in each exposure category, or summary measures of exposure |
|  |  | *Cross-sectional study—*Report numbers of outcome events or summary measures |
| Main results | 16 | (*a*) Give unadjusted estimates and, if applicable, confounder-adjusted estimates and their precision (eg, 95% confidence interval). Make clear which confounders were adjusted for and why they were included  Table 2 |
|  |  | (*b*) Report category boundaries when continuous variables were categorized  N/A |
|  |  | (*c*) If relevant, consider translating estimates of relative risk into absolute risk for a meaningful time period  N/A |
| Other analyses | 17 | Report other analyses done—eg analyses of subgroups and interactions, and sensitivity analyses  Spatial analysis reported in Results p16-17 |
| Discussion | | |
| Key results | 18 | Summarise key results with reference to study objectives  Discussion esp. p17-18; Conclusions |
| Limitations | 19 | Discuss limitations of the study, taking into account sources of potential bias or imprecision. Discuss both direction and magnitude of any potential bias  Limitations of haplotype resolution - Discussion p19  Limitations of spatial analysis – Discussion p20  Limitations of haplotype-symptom associations – Discussion p20 |
| Interpretation | 20 | Give a cautious overall interpretation of results considering objectives, limitations, multiplicity of analyses, results from similar studies, and other relevant evidence  Discussion & conclusion |
| Generalisability | 21 | Discuss the generalisability (external validity) of the study results  This is a descriptive study of a pathogen population circulating in a particular location and time period. This is compared to similar studies in other locations (Discussion para 1 and last para) but is not generalisable per se. |
| Other information | | |
| Funding | 22 | Give the source of funding and the role of the funders for the present study and, if applicable, for the original study on which the present article is based  Acknowledgements, p22 |

*Give information separately for cases and controls in case-control studies and, if applicable, for exposed and unexposed groups in cohort and cross-sectional studies.

**Note:** An Explanation and Elaboration article discusses each checklist item and gives methodological background and published examples of transparent reporting. The STROBE checklist is best used in conjunction with this article (freely available on the Web sites of PLoS Medicine at http://www.plosmedicine.org/, Annals of Internal Medicine at http://www.annals.org/, and Epidemiology at http://www.epidem.com/). Information on the STROBE Initiative is available at www.strobe-statement.org.
