# Supplementary material for: Temporal Fluctuation of Multidrug Resistant Salmonella Typhi Haplotypes in the Mekong River Delta Region of Vietnam
Source: PLoS Negl Trop Dis. 2011 Jan 4;5(1):e929. doi: 10.1371/journal.pntd.0000929 (PMC3014949; doi:10.1371/journal.pntd.0000929)
Supplement: Translation S1 — Translation of the abstract into Vietnamese by Nga Tran Vu Thieu. (0.09 MB RTF) [file pntd.0000929.s003.rtf]

Tóm tắt
Nội dung chính
Sốt  thương hàn, bệnh gây ra do Salmonella Typhi (S. Typhi), vẫn là vấn đề sức khỏe cộng đồng cần được quan tâm tại một số vùng trên Việt Nam như Đồng Bằng Sông Cửu Long. S. Typhi thường đa kháng với các kháng sinh thế hệ thứ nhất và thường giảm nhạy với fluoroquinolones, là  họ kháng sinh phổ biến nhất được dùng để điều trị sốt thương hàn. Chúng tôi sử dụng phương pháp so sánh điểm đa hình (SNP) để phân tích quan hệ di truyền của các chủng S. Typhi phân lập được từ 264 bệnh nhân sốt thương hàn ở Đồng Bằng Sông Cửu Long trong khoảng thời gian 2004 – 2005.
Kết quả chính
91% các chủng S. Typhi phân lập được trong nghiên cứu đều chỉ thuộc một phức hợp dòng (clonal complex) duy nhất đó là S. Typhi H58 haplogroup. Kết quả thu được nhất quán với trận dịch sốt thương hàn ở Đồng Bằng Sông Cửu Long vào năm 2004, gây ra do duy  nhất một dòng S. Typhi H58-E2, và số ca được ghi nhận trong trận dịch năm 2004 này cao hơn 50% so với trong năm 2005. Các chủng S. Typhi, và những trường hợp sốt thương hàn do dòng H58-E2 đều được ghi nhận là mang tính chất địa phương. Bệnh nhân nhiễm S. Typhi H58-E2 có tỉ lệ bị táo bón cao hơn (OR 2.6), nhưng tỉ lệ bị tiệu chảy và nhức đầu lại thấp hơn (OR 0.46; OR 0.54) so với khi nhiễm các dòng S. Typhi khác. Đa kháng thuốc phổ biến ở các chủng thuộc dòng H58-C đã được công bố nhưng điều này không đươc ghi nhận đối với H58-E2. Mặc dầu vậy nhưng tất cả các chủng S. Typhi H58 đều kháng Nalidixic acid, và cùng mang một đột biến đồng nhất liên quan đến kháng thuốc trên gene gyrA.
Kết luận quan trọng
Halogroup H58 cũng chiếm ưu thế trong các quần thể S. Typhi ở các vùng bệnh khác đã được nghiên cứu trước đây, nhưng các chủng trong nghiên cứu này cho thấy mức độ đồng nhất cao hơn. Ngoài ra, phức hợp dòng chiếm ưu thế này (H58-C, -E1, -E2) chưa từng được miêu tả ở bất kì chủng S. Typhi nào phân lập ngoài Việt Nam. Sự khác biệt về đặc điểm lâm sàng trên bệnh nhân nhiễm S. Typhi H58-E2 so với nhiễm H58-C là một ghi nhận độc đáo về cách thức biểu hiện bệnh gây ra bởi hai dòng S. Typhi lân cận gây bệnh trên cùng một quần thể người bệnh đồng nhất. Dữ liệu thu được của nghiên cứu này cung cấp một cái nhìn sâu sắc đến dịch tễ học phân tử của S. Typhi ở miền Nam Việt Nam.
